# Utilization and Experiences of Using Quit Now, a Nicotine and Tobacco Smoking Cessation Website: Thematic Analysis

**DOI:** 10.2196/55592

**Published:** 2025-03-04

**Authors:** Tala Salaheddin, Ramona H Sharma, Marcela Fajardo, Cameron Panter, Lauren De Souza, Sheila Kanyevu Matano, Laura Struik

**Affiliations:** 1 Doctor of Medicine Program Faculty of Medicine University of British Columbia Vancouver, BC Canada; 2 School of Social Work Faculty of Health and Social Development University of British Columbia Okanagan Kelowna, BC Canada; 3 BC Lung Foundation Vancouver, BC Canada; 4 School of Nursing Faculty of Health and Social Development University of British Columbia Okanagan Kelowna, BC Canada

**Keywords:** smoking cessation, user experiences, nicotine, vaping, web-based, Google Analytics, thematic analysis, digital health, nicotine replacement therapy, quit attempts, tobacco, British Columbia, behavioral support, pharmacotherapy, qualitative interview, cessation support, QuitNow, mobile health, mHealth, intervention

## Abstract

**Background:**

British Columbia residents have access to a program called QuitNow that provides behavioral support and information about pharmacotherapy to nicotine and tobacco users. Web- or computer-based smoking cessation programs have been shown to yield an abstinence rate about 1.5 times higher when compared to a control. Although quantitative evidence reveals significant promise for web-based services like QuitNow, there is very little qualitative evidence available. Understanding website utilization and the experiences of end users is key to contextualizing the effectiveness of web-based cessation services and providing directions for enhancing these services.

**Objective:**

This qualitative interview study aims to delve into users’ utilization and experiences of QuitNow, which is supplemented by Google Analytics data.

**Methods:**

We interviewed 10 QuitNow users using semistructured interviews to understand what they liked the most and the least about QuitNow. We transcribed these interviews and conducted an inductive thematic analysis using NVivo (QSR International) software to extract common themes about user experiences. We also gathered utilization metrics via Google Analytics (n=13,856 users) to understand which aspects of QuitNow were used the most and which were used the least during the study period.

**Results:**

Thematic analysis yielded four major themes: (1) barriers to information access reduce opportunities to take action, (2) lack of clarity around pharmacological options is discouraging, (3) hearing from others is an important part of the journey, and (4) recognizing own agency throughout the quit process. These themes provided context and support for the Google Analytics data, which showed that end user activity, measured by indicators such as page views and average time spent on each page, was highest on pages about how to quit (10,393 page views), pharmacology information (1999 page views), and the community forum (11,560 page views).

**Conclusions:**

Results of this study point to several important implications for improving the website, as well as directions for enhancing cessation support services in general.

## Introduction

Smoking, a leading cause of preventable deaths worldwide, poses a significant challenge for individuals attempting to quit. The prevalence of current cigarette smoking among Canadians aged 15 years and older in 2022 was 10.9%, unchanged since 2020, but decreased from 12% in 2019 [1,2]. Notably, in 2020, British Columbia had the lowest smoking rate among Canadian provinces, at 7.7% [3], and continues to boast the lowest smoking rate of 8.7% among all Canadian provinces in 2022. Similarly, vaping rates are also lowest in British Columbia, with a past 30-day vaping rate of 4.7% in those aged 15 years and older [2]. This achievement can be attributed in part to the British Columbia government’s initiatives, such as the British Columbia Smoking Cessation Program, including QuitNow, which supports residents in covering the costs of Nicotine Replacement Therapy (NRT) and smoking cessation prescription drugs and provides those attempting to quit a variety of nicotine products with access to community and expert coaches.

Nearly two-thirds of Canadians who smoke express intentions to quit in the next 6 months and half report that they have tried to quit in the past year [4]. Of Canadians aged 15 years and older who report daily vaping, almost half (42.4%) have made at least one quit attempt in the last 12 months [2]. However, unaided quit attempts have a very low success rate, with fewer than 5% achieving abstinence for 12 months or more [5]. To achieve meaningful reductions in tobacco-related morbidity and mortality, we must increase both the number of individuals who attempt to quit and the success rate of those attempts. Guidelines recommend combining pharmacotherapies with behavioral interventions to enhance the effectiveness of smoking cessation attempts [6,7]. Web-based programs have emerged as a valuable tool in this context. A meta-analysis of 22 randomized controlled trials has demonstrated the clinical efficacy of web-based smoking cessation programs for adults who smoke [8]. This study included 29,549 participants and revealed that web- or computer-based smoking cessation programs yielded an abstinence rate about 1.5 times higher than the control group, and this was observed at both 6 and 12 months of follow-up [8].

Today, 5.18 billion people (two-thirds of the global population) are currently connected to the World Wide Web [9]. From 2000 to 2023, web usage grew more than 1000%, and this number is predicted to continue to grow [10,11]. In recent years, the expansion of web-based systems in Canadian health care has notably enhanced service delivery and patient engagement. For instance, the number of Canadian primary care physicians using electronic medical records has risen from 73% in 2015 to 93% in 2022, facilitating better patient care through improved information sharing between providers, such as lab results and referrals [12]. The COVID-19 pandemic has also led to a rise in the usage of web-based health care, consequently shifting nicotine usage and cessation needs. As the number of global web users grows and the internet becomes increasingly embedded in health care systems, web-based cessation programs are poised to become a more valuable public health strategy [13]. Ensuring the quality and effectiveness of these programs will be crucial in providing accessible support to nicotine and tobacco users worldwide.

QuitNow, a free web-based cessation program provided by the British Columbia Lung Foundation on behalf of the Government of British Columbia, offers information on nicotine and its harmful effects, access to an online community, and one-on-one guidance for individuals seeking to quit or reduce tobacco and e-cigarette use [14]. While previous research has explored the overall impact of online cessation support, there is limited knowledge about the in-depth experiences of end users and how their experiences contextualize specific utilization data [15].

Web-based cessation programs, such as QuitNow, are designed to provide tailored support and resources to individuals seeking to quit smoking or using other nicotine products. The effectiveness of these platforms is heavily dependent on how users interact with the content and features provided. For instance, user engagement metrics, such as page views, time spent on specific sections, and interaction with multimedia elements, offer valuable insights into which aspects of the platform are most effective or need improvement. By analyzing these interactions, developers can make data-driven decisions to enhance the platform’s usability, accessibility, and overall impact.

This study aims to address the gap in knowledge by investigating the in-depth experiences of individuals who used QuitNow as a quit aid and examining how these experiences are reflected in utilization data, such as that provided by Google Analytics. By understanding end user experiences better, we can tailor our efforts to support British Columbia residents in reducing nicotine and tobacco use, thereby improving overall health outcomes.

## Methods

### Participants

Interview data from 10 individuals who had used QuitNow were included in this study. These participants were part of a larger study that aimed to understand the perceptions and experiences of QuitNow among individuals who use a variety of nicotine products. For this study, we examined data only from participants who reported using QuitNow to support their cessation (vs participants who had not used QuitNow) and included participants who had recently quit or were motivated to quit, either smoked, vaped, or both, were aged 16 years or older, lived in British Columbia, and could communicate in English. The recruitment procedures and inclusion and exclusion criteria are described in a previous study [16].

Out of the 10 participants, 8 were interested in quitting smoking or vaping, and 2 had recently quit (one quit smoking, one quit vaping). The mean age of participants was 34.5 (SD 17-55) years; 70% (7/10) were male, 50% (5/10) were Caucasian, 80% (8/10) smoked cigarettes, and 60% (6/10) used e-cigarettes. Most individuals who used traditional cigarettes (7/8, 88%) had been smoking for over 5 years, while 100% (6/6) of individuals who used e-cigarettes had been vaping for less than 5 years. Of all participants, 40% (4/10) only used traditional cigarettes, 30% (3/10) used both traditional and e-cigarettes, and the remaining 30% (3/10) had only used e-cigarettes.

### Data Collection

All consenting participants filled out a brief demographic survey encompassing personal characteristics (eg, gender, age) and information about their smoking and vaping history. Participants then engaged in an approximately 60-minute, audio-recorded, semistructured interview via University of British Columbia Zoom. Interviews began with introductions and a reminder about the study’s purpose and procedures. After participants answered questions about how they heard about QuitNow and what motivated them to use QuitNow, they were then asked 5 primary questions, including what they liked about QuitNow, what they did not like about QuitNow, suggestions for improving QuitNow, what aspects outside of QuitNow supported their quit journey, and their “ideal world” vision for how QuitNow could help them with quitting in the context of their lives. After interview completion, each participant received a CAD $50 (US $34.97) electronic gift card to thank them for their contribution to the study. Interview recordings were transcribed for analysis.

### Data Analysis

We conducted an inductive thematic analysis of 10 interview transcripts following guidelines from Willig et al [17]. The interviews were first transcribed verbatim and uploaded to the qualitative data analysis software program NVivo (QSR International). The analysis process began with two researchers (TS and LS) reading the transcripts while making casual observational notes to engage with and familiarize themselves with the data. After a detailed reading of the transcripts, the researchers began to generate codes and systematically label and synthesize segments of the dataset to reduce and organize it. For example, when participants described barriers of the website for supporting their cessation, the researchers inserted these data under the larger code “barriers to quitting,” which then contained specific sublabels for these barriers (eg, “too much text to process;” “can’t access the full site without providing personal details”). We did the same with “facilitators to quitting.” Data saturation was achieved, whereby we found no new interview data emerged to answer our research question after the sixth interview. Following this, the researchers iteratively developed a coding framework using interview data. One author (TS) then sorted associated tagged data segments according to the developed codes, which were revised or added to as new data were collected. The researchers then examined the codes and their associated data and clustered them to construct themes from a well-rounded understanding of the transcripts. For example, when examining codes around website-based barriers and facilitators to quitting, we recognized overall themes. For example, while participants talked about how too much text limited their ability to really take something away from a certain page and apply it in practice, they also talked about how graphic-based information enabled them to learn and apply ideas to facilitate quitting, something that was lacking. Therefore, these codes were grouped together and formed the theme “Barriers to information access reduce opportunities to take action.” These were reviewed and defined to help answer the research question. Author TS then finalized the findings and produced the report, which was reviewed by all other authors for agreement.

### Google Analytics Data

To understand empirical information on public usage of the QuitNow website and related trends (eg, which pages on QuitNow were most frequently navigated to, the average duration of time spent per page), Google Analytics data from the period during which participant interviews were conducted (March 3-July 6, 2021) were also gathered. Analytics data included information on overall website usage and user reach, along with detailed web page-level metrics capturing typical user behaviors on the site (eg, number of sessions; length of sessions).

### Ethical Considerations

Ethics approval for this study was obtained from the University of British Columbia (Okanagan) Behavioral Research Ethics Board (H21-00145-A006). All participants were required to provide informed consent. Before their interview, participants were provided with an emailed consent form and informed that their participation was voluntary; they then provided written consent via email. Consent to participate and to be audio-recorded was reiterated at the beginning of each interview. In addition, participants were reminded that their participation was entirely voluntary and that they could withdraw from the study at any time. Each participant received a CAD $50 (US $34.97) honorarium to thank them for their contribution to the study. To protect participant identity, participants were given an identification number to anonymize their responses. Personal information such as names and contact details was not included in the dataset and was securely stored separately from the study data. Audio recordings and transcripts were kept in password-protected files, accessible only to the research team. The datasets generated during and analyzed during this study are not publicly available due to the protection of participant identity but deidentified data are available from the senior author (LS) on reasonable request.

## Results

### Qualitative Themes

#### Overview

This study resulted in four major themes to capture the experiences of individuals who used QuitNow to support tobacco and nicotine cessation. These themes include: (1) barriers to information access reduce opportunities to take quit action, (2) lack of clarity around pharmacological options is discouraging, (3) hearing from others is an important part of the journey, and (4) recognition of one’s own agency in the quit process. Some of these themes contain subthemes, which will be elaborated in the following sections.

#### Theme 1: Barriers to Information Access Reduce Opportunities to Take Action

##### Sharing Personal Information to Access Information Is a Barrier

In this study, a number of participants expressed reluctance and discontent with the process of creating an account and sharing personal information, citing various reasons for their reservations. Some of these included discussions on user privacy and even references to other websites that allow users to be anonymous and thus more genuine. For example, participant 49 (male, an individual who smokes) said:

one of the benefits of Reddit is [that it] is anonymous. So people are naturally inclined to be more open.

Participant 51 (female, an individual who smokes) believes that:

[the website] could be a little bit less intrusive with respect to personal information, then that might be helpful (…) I mean, there’s so many issues with identity theft and security.

Participant 17 (male, an individual who vapes) even explicitly stated that:

what kept [them] from really using [the website] is the fact that you have to kind of create an account.

In fact, one participant (P73, male, an individual who dual uses) even wondered:

what if somebody is not ready to sign up yet, but they’re interested?

This highlights that web designers should reduce barriers to accessing the website. To address these issues, more than one participant suggested having a “guest function.” In describing this feature, participant 73 (male, an individual who dual uses) said:

like you don’t have to, like log in and have like a name, but you can like be a guest and look at all the other stuff and here are all the options that are available after you make your quit plan.

##### Lack of Content Clarity and Usability Is a Barrier

Another barrier to consider is that commonly used formats, such as primarily online-based and text-heavy ones, may not support effective and inclusive engagement for all individuals. Therefore, alternative formats should be considered. These include non–web-based platforms, as well as more accessible online platforms that are user-friendly, incorporate visuals, and use clear and simple language when internet access is not a barrier. Specifically, participant 86 (female, individual who dual uses) states that:

a lot of people in [place] don’t have internet either so it’s like you could tell them about it but they can’t access it so maybe a pamphlet would be better.

To minimize challenges associated with the current online platform, participants recommended reducing the amount of text per page and allocating a tab on the website that would allow immediate access when the cravings hit. To illustrate these ideas, participant 49 (male, an individual who smokes) suggested:

point form [and] breaking down to one or two sentences per paragraph.

Participant 86 (female, individual who dual uses) even brought up how cravings last around 3-5 minutes and the importance of having an easily accessible tab that gives:

suggestions about things that you can do […] that might be more productive so that when that craving hits then maybe it’s like you have a list of like things to quickly go and do

Not only can the text be made more comprehensible, but more visual components can be added, as alluded to by some individuals. Participant 49 (male, individual who smokes) said:

The information could be supplemented with images or replaced by images” while another participant agrees and describes visiting pages that have a “wall of information and if that can be divided up into more of a visual infographic [then] it would just bring the point across better.

#### Theme 2: Lack of Clarity Around Pharmacological Options Is Discouraging

When talking about NRT, participants were pleased that it is provided free of charge through the British Columbia Smoking Cessation Program via Pharmacare. Participant 34 (male, individual who smokes) said:

It’s a nice feature to have the provincial government sponsoring that.

However, while they appreciated its availability, they also highlighted the lack of information and clarity about this program on the QuitNow website. Many participants were unaware of the availability of free NRT, how the program functions, and how much NRT they could obtain for free. For example, participant 34 (male, an individual who smokes) said:

I mean, I go to the grocery store and look at the patch on the shelf. And it's like, 50, $60, and, you know, for a week, and you're like, yeah, I spend that much on cigarettes or whatever. So it’s a no.

Even when some participants were aware of the program, they still faced difficulties accessing more NRT when needed and expressed frustration due to the lack of clear guidelines on obtaining and using the free NRT.

The only frustrating, I think, is like I guess be more information about the nicotine replacement therapy program that’s offered for free in B.C. because like I ran out of mine so I couldn’t get anymore and so I think like I didn’t know that until I went back to get more and they said I was out and like you could only get so much in 3 months. So maybe just some more information about like specifically how to get that because I had to tell a lot of people about it too so like how to get it, how much you can get for free.

participant 86, female, an individual who dual uses

#### Theme 3: Hearing From Others Is an Important Part of the Journey

##### Hearing From Others Who Smoke

When describing what helped them during their quit journey, participants highlighted the importance of hearing from both current and former smokers who had successfully quit. Participant 34 (male, an individual who smokes) shared:

I really liked the inspirational stories about the other people quitting. I think that was really positive. Especially when you see people have [smoked] for years, and then they’ve given up and they're happy.

Similarly, when reflecting on the community forum, Participant 51 (female, an individual who smokes) emphasized the value of going through the process with others:

I am really not alone in this journey, right. And also, when people share their experiences, it just helps us connect better. Connect better with the situation, challenges, and success.

Participant 60 echoed this sentiment, stating:

It shows you that it’s not just you. A lot of other people are trying to quit, and also using the app and trying hard.

##### Communication From Professionals

Participants emphasized the importance of being able to connect with professionals, such as quit coaches, during their quit journey. These experts provided not only practical advice but also emotional support, offering tailored guidance that complemented the information on the QuitNow website. Participant 60 (male, individual who vapes) shared that:

what was really…helpful is [that] you can communicate with an agent [..] whenever you need someone to help you, whenever you have questions, whenever you anything, you can just one click.

Beyond answering technical questions, professional interaction also helped users feel supported in moments of uncertainty or high cravings, reinforcing their motivation to quit. Participants appreciated the immediacy and personalization of the support, which made them feel they were not navigating the process alone. Some suggested that future versions of QuitNow could include more proactive communication, like scheduled check-ins or reminders, to keep users engaged and motivated throughout their quit journey.

#### Theme 4: Recognizing Own Agency Throughout the Quit Process

While participants provided their insights and experiences with using QuitNow to aid with their cessation, they described how important it was to recognize their own agency in the quit process. When answering the question about what the biggest factor in reducing or quitting was, one participant said:

I think it’s myself

participant 51, female, an individual who smokes

Building on this, participants described how support programs, like online cessation websites, are valuable tools that can support their path toward a smoke-free life, but that the support offered must parallel the smoker’s determination to quit. This is exemplified by the following participant statement:

But until you want to [quit], I don’t know if you can [quit]. I mean, if we change it around and…we were talking about a fitness website, well, the fitness website isn’t going to get you fit. It's going to give you a whole bunch of ways to get fit [and] reasons to get fit, lots of help, but it's up to you. I don't see a quit smoking website any different than that…there’s no magic button that you're going to click on. And all of a sudden, you're not going to have no desire to smoke

P39, male, individual who smokes

### Exploring Theme Interrelations

An examination of how the identified themes interrelate reveals a complex and interconnected landscape of user experiences with QuitNow. For instance, “Barriers to information access” (Theme 1) directly influence “Lack of clarity around pharmacological options” (Theme 2). When users face difficulties in accessing or understanding information due to privacy concerns or inadequate dissemination strategies, their ability to fully grasp and use pharmacological aids, such as NRT, is compromised. This lack of clarity can diminish the perceived value of such options, thereby impacting users’ motivation to engage with them. In addition, “Recognition of own agency” (Theme 4) underscores the significance of individual determination in the quit process, which can be either supported or undermined by the other themes. For example, overcoming barriers to access (Theme 1) and achieving clarity on pharmacological options (Theme 2) can empower users to take more control over their quit journey, reinforcing their sense of agency. Conversely, insufficient support and unclear information may erode this sense of agency, demonstrating how these themes collectively shape and influence the quit process. By understanding these interconnections, we gain a more nuanced perspective on how different factors interplay to affect user experiences and outcomes in tobacco and nicotine cessation.

### Google Analytics Findings

#### User Characteristics

Over the period of study, QuitNow’s web platform was accessed by a total of 13,856 clients across British Columbia. Of these, 1723 were active users of QuitNow digital interventions, a collection of web-delivered services designed to support nicotine cessation goals. By contrast, the provincial Quitline hosted at QuitNow supported a total of 1178 clients over this same period, although it must be noted that we are unable to test for overlap between clientele accessing digital interventions and those contacting the provincial Quitline for support. The QuitNow web platform tended to serve younger clients than the traditional Quitline service, with an average age of 32.8 over the period of study relative to 54.9 among Quitline callers. Both service lines saw a similar distribution of genders, serving approximately 58% female, 40% male, and 2% nonbinary clients.

#### User Behavior

Of the 66 unique pages that make up the quitnow.ca platform, Tables 1 and 2 identify the 10 most- and the 5 least-trafficked pages during our study period, respectively. Also included are the total unique user counts over the study period for each page, the bounce rate (the percentage of users who only view one page and leave without any interaction, or whose view lasts less than 10 s), average time (how long a user spent browsing a particular page), average session time (how long a user spent on the website), and pages per session (how many pages a user visited during one session).

Beyond basic administrative pages such as the homepage, account creation, and sign-up pages, the most popular features of the QuitNow website tended to be community- and information-driven. The community forum and support pages both feature among the 10 most trafficked, with visitors to the community forum also averaging the most time on site per session. Information-sharing pages also feature heavily in the top-10 list, including “Get started now,” “What does smoking cost,” and “Medications can help.” Less popular pages of the site highlight ways to distract oneself from nicotine cravings; “Get active,” “Change your routine,” and “Stay busy” are all among the 5 least-trafficked pages on the site.

In Table 1, a list of the top 10 most visited pages on the QuitNow website between March 3 and July 6, 2021, is given, highlights user engagement metrics such as page views, number of unique users, bounce rates, average time spent on each page, average session duration, and pages visited per session. These metrics were analyzed as part of our qualitative study exploring user experiences and engagement with QuitNow. Key observations include high engagement with the homepage and the community forum, which had significant page views and longer average time spent, while pages like “Join” and “Get started now” experienced higher bounce rates, suggesting opportunities for improved user retention. This data provides insights into how users interact with the site and which content resonates most with them.

**Table 1:** Top 10 pages visited by QuitNow users from March 3 to July 6, 2021.

Page	Topic	Page views	Users	Bounce rate	Average time	Average. session time	Pages per session
Homepage	The introduction page (users logged out)	22,719	9171	33%	00:00:39	00:02:54	1.42
Forum	Community forum for peer and coach support where users can share their experience	11,560	1918	59%	00:04:44	00:02:52	2.07
Quit Plan	Personalized guide for quitting when the users are logged in	10,393	1904	50%	00:02:48	00:05:49	2.84
Join	Web form to join the program	8107	7217	75%	00:01:36	00:01:26	2.04
Sign-in	Page to sign-in into the profile	7655	1326	10%	00:00:19	00:05:57	4.2
Get started now	Explanation of the program, why to create an account and steps	2553	1667	85%	00:01:19	00:00:38	1.36
What does smoking cost?	Interactive calculator to see how much money one spends	2317	1467	63%	00:03:37	00:01:31	1.41
Medications can help	Guide and description of the available medications in the province	1999	1159	75%	00:02:19	00:01:45	1.76
Community and support	Access to the services QuitNow provides to the community	1615	814	59%	00:00:43	00:01:11	2.3
Quitting	More information about stages of the quit process, from thinking about to preparing, to quitting, to staying quit.	1389	799	35%	00:00:33	00:02:38	3.51

In Table 2, the metrics were analyzed as part of our qualitative study exploring user experiences and engagement with QuitNow. These pages cover niche topics such as staying active, scheduling calls with Quit Coaches, supporting loved ones on their quit journey, coping with cravings, and finding distractions. Despite low user numbers, some pages—such as “Get Active” and “Supporting Loved One”—had notably high pages per session and long average session times, indicating deep engagement by the few who visited them. Conversely, other pages like “Stay Busy” and “Change Your Routine” experienced higher bounce rates and shorter time spent, suggesting potential areas for improvement in content or accessibility. This data helps identify underused resources and guides strategies for increasing visibility and user interaction.

**Table 2:** Least 5 visited pages on the QuitNow website between March 3 and July 6, 2021, focusing on user engagement metrics including number of unique users, bounce rates, average time spent on each page, average session duration, and pages visited per session.

Page	Topic	Users	Bounce rate, %	Average time	Average session time	Pages per session
Get active	The introduction page (users logged out)	107	33	00:01:44	00:05:47	7.33
Schedule a call	Page to schedule a call with one of the Quit Coaches	60	50	00:00:37	00:01:03	3
Supporting loved one	How to support and behave with people who are on their quit journey	52	28	00:00:59	00:07:04	7
Change your routine	Tips to cope with cravings and triggers	65	50	00:00:53	00:03:25	4.25
Stay busy	Advice and recommendations on how to get distracted from cravings	35	50	00:00:30	00:07:52	4.5

## Discussion

### Principal Findings

This study brings forward the end user experiences and utilization metrics of using a web-based cessation program (QuitNow) among individuals who want to quit smoking or vaping. In light of the shifting trends in nicotine and tobacco product use (eg, rise in use of e-cigarettes), demographic behaviors (eg, high vaping prevalence among youth), the rapid rollout of new digital technologies, and the high use of digital platforms, these findings provide important directions for optimizing such platforms. In particular, the findings bring forward important contextual factors that are unique to today compared with traditional approaches.

As the availability and use of digital platforms continue to rise and as nicotine users, particularly younger demographics (teens and young adults), navigate an increasing number of online platforms, the expectation for engaging and accessible content delivery becomes paramount. As indicated by individuals who use QuitNow, engaging formats not only capture attention more effectively but also contribute to a more relatable and memorable learning experience, which are crucial elements in supporting individuals on their cessation journeys [18]. A lack of information delivered in a variety of accessible, engaging, and visual formats may thus explain why the QuitNow Google Analytics data indicate that end users are not engaging with several pages focused on ways to distract from cravings despite our qualitative findings indicating end users’ desire for rapid access to tips for dealing with cravings. Therefore, digital cessation supports must shed their traditional reliance on text-heavy formats and instead, leverage innovative and engaging multimedia elements, such as video testimonials, interactive graphics, podcasts, and opportunities to share on social media. Indeed, existing research highlights that, in the context of nicotine cessation, where motivation and sustained commitment are essential, a visual and interactive presentation of information serves as a powerful catalyst for positive behavioral change [19]. In addition to boosting engagement, interactive modalities may also foster a deeper connection with users by catering to varied learning preferences and thus enhancing accessibility [20]. Therefore, our findings emphasize that the effectiveness of nicotine cessation support websites relies not only on the quality of their content but also on the delivery methods they use.

The qualitative findings also indicate that information on pharmacotherapy options for quitting is desired, and the Google Analytics data indicate that information on pharmacotherapy options ranks among the top 10 pages visited on QuitNow. Pharmacotherapy is vital for individuals seeking to quit smoking and vaping, primarily addressing the physiological aspects of nicotine addiction. Approved pharmacotherapy for smoking cessation includes NRT products and several prescription medications. Research has consistently highlighted the effectiveness of pharmacotherapy, with some research indicating that those who use NRT or prescription medications are up to 2-3 times more likely to quit than those who attempt cessation without pharmacotherapy [21-24]. NRT offers a controlled and regulated supply of nicotine without exposing individuals to the more than 7000 chemicals found in cigarette smoke or vape aerosol [21]. This controlled supply of nicotine helps reduce withdrawal symptoms, such as cravings and irritability, making quitting more manageable and significantly reducing the risk of relapse [25]. The combined use of a long- and short-acting form of NRT (eg, the nicotine patch and the nicotine gum) is considered the most effective method of NRT use [21]. It is particularly effective for those with more severe addictions or those who have not succeeded with single-form NRT [26]. This evidence-based approach, supported by extensive scientific research, underscores the significance of NRT, including combination therapies, as indispensable tools in the cessation process. The prescription medications for cessation include varenicline (brand name Champix) and bupropion (brand name Zyban). Varenicline helps reduce nicotine cravings and reduces the satisfaction one gets from smoking or vaping. Bupropion helps reduce nicotine cravings and withdrawal symptoms. Varenicline is one of the pharmacological tools most likely to help people quit smoking, with 12-16 of every 100 individuals quitting smoking using varenicline compared to 6 of 100 who did not use any medicine. After using bupropion, 9 of 100 people are likely to quit [27].

The British Columbia Smoking Cessation Program has been a commendable initiative aimed at supporting British Columbia residents in their efforts to quit smoking. The program provides free or low-cost access to NRT products, such as gum, patches, and lozenges, for eligible participants, significantly reducing financial barriers to quitting. In the most recent evaluation of the program, data showed that it had provided coverage for more than 25% of British Columbia residents who smoke. From September 30, 2011, to March 31, 2020, over 354,900 patients received smoking cessation aids (294,700 for nicotine gum, inhaler, lozenge, or patches, and 118,700 for bupropion or varenicline), with the British Columbia Ministry of Health investing approximately CAD $112.2 (US $78.47) million for product coverage. While the program has played a valuable role in aiding smoking cessation, it is essential to acknowledge that there is room for improvement. Currently, the program does not support residents in vaping cessation. In addition, the program does not allow residents to access combination NRT. While current policy allows switching from one product to another during treatment, only one of either NRTs or prescription medication is covered for 12 weeks each calendar year, which may not be enough for residents looking to quit. As well, 12 weeks of treatment is often not sufficient to achieve abstinence and prevent relapse. As a result of this limitation, some residents reported paying out of pocket for the products they used. Of over 4000 residents surveyed for the evaluation, a third of respondents (35%) indicated they had out-of-pocket expenses for other products or activities to help them quit smoking. The average monthly costs associated with these cessation products or activities were CAD $102 (US $71.34) and ranged from CAD $1 to $2000 (US $0.70 to $1398.75) a month. In light of our qualitative findings, the program must adapt to better support residents considering the evolving landscape of nicotine addiction, including the rising prevalence of vaping and emerging research on more effective cessation strategies. Collaboration between the government, community stakeholders, providers, and pharmacies is crucial for enhancing the program’s reach and efficacy, ensuring it remains a robust and relevant resource in promoting a smoke-and-vape-free British Columbia. Addressing these needs is critical, as pharmacotherapy is a substantial component of a successful quit trajectory.

Our findings also indicate how incorporating social support on cessation support websites is crucial to providing end users with a sense of community, encouragement, and shared experiences. Existing research mirrors this; peer support has been shown to positively impact cessation outcomes by fostering motivation, accountability, and the exchange of coping strategies [28]. Similar to findings from a previous study on QuitNow, implementing and enhancing features, such as discussion forums, live chat options, or virtual support groups provide end users with the ability to connect, share challenges, and celebrate successes [29]. In addition, integrating social media elements may allow users to easily share their progress, garnering support from friends and family beyond the platform. By fostering a supportive online community, cessation websites like QuitNow can enhance user engagement and ultimately contribute to sustained success with respect to user quit journeys, which is emphasized in our qualitative findings, and further by the Google analytics data indicating that the community forum and support ranked among the top 10 visited pages.

Finally, our findings illuminate the importance of a collaborative and synergistic nature between the quitting individual’s personal agency and the resources offered by a cessation platform, like QuitNow. As such, it is crucial for cessation websites to embody a commitment to foster a collaborative relationship with end users in their quit journey versus embodying a commitment to being the “silver bullet”—the perfect solution that removes all responsibility from the person who wants to quit. In this regard, websites must function as allies in this process by providing a structured and informed environment that catalyzes the user’s cessation journey (ie, by including personalized tools, expert guidance, and a supportive community). Ultimately, incorporating a collaborative and person-centered lens in website development and delivery can not only acknowledge the significance of personal agency and make users feel more capable but also underscore the role of external support in enhancing the efficacy and sustainability of the quitting process.

### Strengths and Limitations

This study is subject to a few limitations. First, our small sample size may have masked existing themes representative of the broader population of QuitNow users. In addition, as we did not explore thematic differences in cessation needs based on gender, ethnicity, or other demographic markers, there may be identity-based nuances in QuitNow user needs that our data did not capture. Participant accounts within qualitative interviews are also subject to recall and social desirability biases, which together with the previous may have influenced which themes were most apparent in the data. Further, our findings are limited by the scope of thematic analytic techniques; coding processes used in the thematic analysis are subjective and open to interpretation and thus are heavily dependent on researcher judgment and participant accounts [30]. Next, our findings are also characterized by limitations based on geographic and temporal contexts. As participants were solely from British Columbia, Canada, participant experiences may not be representative of settings beyond British Columbia. In addition, data were collected during the COVID-19 pandemic (May-August 2021) and since then, changing health and sociocultural contexts and policies may have reshaped experiences and cessation needs of QuitNow and general nicotine users [16]. Finally, the experiences of users were also based on which interface (web vs mobile) they used to access the QuitNow website, which may have influenced their opinions on the site.

Despite these limitations, our study is characterized by unique strengths. First, our use of thematic analysis allowed us to explore both apparent and latent content within user accounts, thus allowing us to capture explicit as well as underlying meanings within the data. In addition, supplementing qualitative themes with web analytics strengthened the analysis by combining subjective insights with quantitative metrics, enhancing the comprehensiveness and credibility of our findings. This dual approach provided a nuanced understanding of user behavior on the QuitNow website and continues to facilitate informed decision-making for targeted content delivery improvements. Finally, collecting rich interview data over months allowed us to capture nuanced, as well as consistent participant viewpoints in the context of an ever-changing pandemic landscape in real time.

### Conclusion

In conclusion, our study on QuitNow user experiences and utilization, supplemented by Google Analytics data, reveals important insights. Integrating our qualitative findings with usage patterns provides clear directions for program enhancement: overcoming information barriers, refining pharmacological information presentation, fostering community support, and incorporating creative motivational elements. Future research should investigate user preferences in a larger and more demographically diverse sample to ensure these findings are applicable to the wider population. However, these insights offer practical strategies to optimize QuitNow and, more broadly, advance the effectiveness of web-based cessation services, contributing to improved public health outcomes.

## Abbreviations

NRT: nicotine replacement therapy

## Data Availability

The datasets generated during and/or analyzed during this study are not publicly available due to the protection of participant identity but deidentified data are available from the senior author (LS) on reasonable request.
